# Impact of Vaccination on Distribution of T Cell Subsets in Antiretroviral-Treated HIV-Infected Children

**DOI:** 10.1155/2017/5729639

**Published:** 2017-06-12

**Authors:** Premrutai Thitilertdecha, Ladawan Khowawisetsut, Palanee Ammaranond, Poonsin Poungpairoj, Varangkana Tantithavorn, Nattawat Onlamoon

**Affiliations:** ^1^Department of Research and Development, Faculty of Medicine Siriraj Hospital, Mahidol University, Bangkok, Thailand; ^2^Department of Parasitology, Faculty of Medicine Siriraj Hospital, Mahidol University, Bangkok, Thailand; ^3^Department of Transfusion Medicine, Faculty of Allied Health Sciences, Chulalongkorn University, Bangkok, Thailand

## Abstract

Antiretroviral therapy (ART) is generally prescribed to patients with human immunodeficiency virus (HIV) infection with vaccination introduced to prevent disease complications. However, little is known about the influence of immunization on T cell subsets' distribution during the course of infection. This study aims to identify the impact of viral replication and immunization on naïve, effector, effector memory, and central memory T cell subpopulations in ART-treated HIV-infected children. Fifty patients were recruited and injected intramuscularly with influenza A (H1N1) 2009 vaccine on the day of enrollment (day 0) and day 28. Blood samples were collected for pre- and postvaccination on days 0 and 56 for analyzing T cell phenotypes by flow cytometry. Phenotypes of all T cell subsets remained the same after vaccination, except for a reduction in effector CD8^+^ T cells. Moreover, T cell subsets from patients with controllable viral load showed similar patterns to those with virological failure. Absolute CD4 count was also found to have a positive relationship with naïve CD4^+^ and CD8^+^ T cells. In conclusion, vaccination and viral replication have a little effect on the distribution of T cell subpopulations. The CD4 count can be used for prediction of naïve T cell level in HIV-infected patients responding to ART.

## 1. Introduction

Disease progression of human immunodeficiency virus (HIV) infection can be observed through changes in the numbers of CD4^+^ and CD8^+^ T cells. Depletion of CD4^+^ T cells occurs throughout three stages of HIV infection (i.e., acute infection, clinical latency, and acquired immune deficiency syndrome (AIDS)), whereas CD8^+^ T cells potentially increase in the first stage and remain during the second stage before depleting in the final stage [1]. Furthermore, monitoring a reduction in naïve T cell from both CD4^+^ and CD8^+^ populations together with an elevation of memory CD8^+^ T cells was useful to determine the disease progression in both HIV-infected adult patients [2] and HIV-infected children [3].

Antiretroviral therapy (ART) is normally used to suppress viral replication in HIV-infected patients whose CD4 count is consequently increased. Pakker et al. confirmed this increase in CD4^+^ T cells by finding that CD4^+^ and memory CD8^+^ T cells were significantly increased in the patients after receiving a highly active ART (HAART) through a redistribution of T cell subsets [4]. Plana et al. also studied HAART-treated patients and found increases in naïve and memory CD4^+^ T cell as well as a decrease in CD8^+^ T cells, suggesting that the earlier the treatment begins, the faster the T cell subset normalization is [5].

Although HAART is very effective at reducing viral load to an undetectable level, the immunological function does not fully recover to pre-HIV levels. Immunocompromised individuals, therefore, still have much higher chances of infection by other pathogenic viruses (e.g., influenza virus) and experience worse symptoms compared to healthy people. Immunization is then given to HIV-infected individuals to prevent severe complications; however, there is evidence showing that vaccination may also adversely affect the immunological status of HIV-infected people. Glesby et al. reported a decrease in CD4^+^ T cells led by influenza immunization [6], and Tasker et al. found the same significant reduction in CD4^+^ T cells in patients, 3 months after receiving a single shot [7]. Several publications have showed contradictory results, indicating that CD4^+^ T cells of patients injected with influenza vaccine had no significant change [8–11]. The influence of influenza immunization on CD4^+^ T cells in HIV-infected patients thus remains controversial.

This study primarily aimed to pinpoint effects of immunization and viral replication on T cell distribution of both CD4^+^ and CD8^+^ T cells together with their subsets (i.e., naïve, effector, effector memory (Tem), and central memory (Tcm) cells) in ART-treated HIV-infected children. The study secondarily purposed to observe a relationship between the classical CD4 and CD8 counts with each T cell subset's frequency.

## 2. Materials and Methods

### 2.1. Study Population, Immunization, and Sample Collection

Fifty HIV-infected children aged between 6 months and 18 years old receiving ART at the Faculty of Medicine Siriraj Hospital, Mahidol University, Bangkok, Thailand, were recruited for the study. The Institution Review Board (IRB) of the Faculty of Medicine Siriraj Hospital approved the study, and written informed consent and parental consent were obtained from each subject prior to the study.

Two doses of influenza A (H1N1) 2009 vaccine were administered to the patients via an intramuscular route. Five hundred microliters and 250 *μ*L were administered to the patients aged above and below 3 years old, respectively. The first inoculation was given on the day of enrollment (day 0), and the second vaccination was given on day 28. Blood samples were collected twice: one before the first shot and another on 28 days after the second shot (day 56). Blood samples were collected in Vacutainer™ tubes containing either ethylenediaminetetraacetic acid (EDTA) or sodium heparin.

### 2.2. Routine Sample Analysis

Each blood sample was divided into two for analyses. The first analysis was a determination of CD4^+^ and CD8^+^ T cells by a flow cytometer and absolute lymphocyte counts by a routine complete blood count (CBC) test. The second analysis was an examination of HIV viral load by Abbott RealTime HIV-1 using plasma collected from an aliquot of the individual blood sample. The viral load value was then used to separate the patients into two groups, controller and noncontroller. Of the 50 patients, 37 patients who had undetectable viral loads (<40 copies/mL) at the enrollment and after the second vaccination (i.e., 56 days after the enrollment) were classified into the controller group. Thirteen subjects who failed those criteria were categorized into the noncontroller group.

### 2.3. Monoclonal Antibodies and Reagents for Flow Cytometric Analysis

Anti-human monoclonal antibodies (mAbs) and their conjugated fluorochromes including anti-human CD45RA conjugated with fluorescein isothiocyanate (FITC), anti-human CD4 conjugated with peridinin chlorophyll protein (PerCP), and anti-human CD62L conjugated with allophycocyanin (APC) were purchased from BD Biosciences (BDB, San Jose, CA) as well as FACS™ lysing solutions. Anti-human CD3 conjugated with PECy7 and anti-human CD8 conjugated with APC-Cy7 were obtained from BioLegend (San Diego, CA). Anti-human CCR7 conjugated with phycoerythrin (PE) was obtained from R&D Systems (Minneapolis, MN). All reagents were used at concentrations recommended by the manufacturers.

### 2.4. Immunofluorescent Staining Method and Flow Cytometric Analysis

Fifty microliters of an individual blood sample was stained with a mixture of mAbs containing CD45RA-FITC, CCR7-PE, CD4-PerCP, CD3-PECy7, CD62L-APC, and CD8-APC-Cy7 and incubated in the dark at ambient temperature for 15 minutes. Two microliters of FACS lysing solution was then added into the mixture and incubated in the dark at ambient temperature for further 10 minutes before centrifugation at 350*g* and 22°C for 5 minutes. The supernatant was discarded and cell pellets were then resuspended in 2 mL wash buffer (a mixture of phosphate-buffered saline (PBS) and 2% fetal bovine serum). The sample was centrifuged at 350*g* and 22°C for 5 minutes. After centrifugation, the supernatant was discarded and the cell pellets were resuspended in 300 *μ*L freshly prepared PBS containing 1% paraformaldehyde before being subjected to the flow cytometer.

The LSR II flow cytometer with FACSDiva software (BDB, San Jose, CA) was used to analyze the prepared samples with at least 100,000 lymphocytes per sample. Results of naïve, effector, Tem, and Tcm subsets from CD4^+^ and CD8^+^ T cell populations were analyzed using FlowJo software (Tree Star, San Carlos, CA).

### 2.5. Statistical Analysis

Data was expressed as an average ± SD (standard deviation) and compared for statistical difference at *P* < 0.05 using the Mann–Whitney *U* test for evaluation between controller and noncontroller groups. Differences between different markers (CCR7 versus CD62L) and between blood samples before and after immunization were evaluated by using the Wilcoxon signed-rank test. Correlations among cell populations were assessed using the Spearman correlation test and considered to have a statistical correlation at *P* < 0.05.

## 3. Results

### 3.1. Identification of T Cell Subsets

To identify CD4^+^ and CD8^+^ T cell subsets including naïve, effector, Tem, and Tcm cells, two mAb combinations of CD45RA with CD62L and CD45RA with CCR7 have been commonly used. Nonetheless, there is no information concerning a difference of using these two mAb combinations in T cell subset identification. This study then observed the differences in terms of flow cytometric plot and cell number.

**Figure 1:**
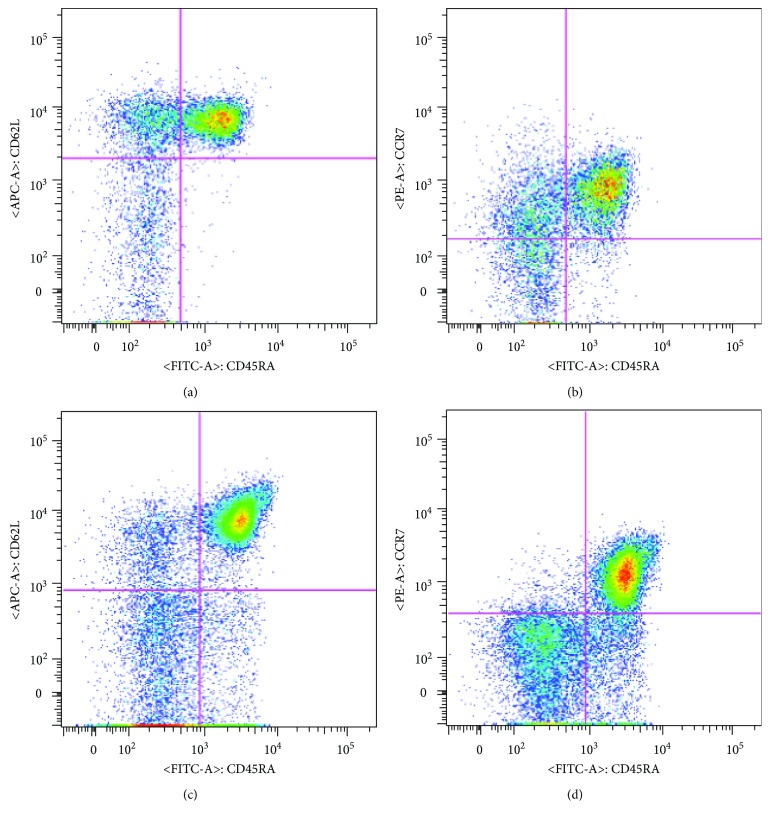
Representative profiles of T cell subsets identified by using CD45RA with CD62L compared to using CD45RA with CCR7 in CD4^+^ T cells (a, b) and in CD8^+^ T cells (c, d).

For phenotypic plot, subpopulations of CD4^+^ T cells (Figures 1(a) and 1(b)) and CD8^+^ T cells (Figures 1(c) and 1(d)) are characterized using the mAb combination of CD45RA with CD62L compared to the one of CD45RA with CCR7. Naïve (CD45RA^+^ CD62L^+^ or CD45RA^+^ CCR7^+^), Tem (CD45RA^−^ CD62L^−^ or CD45RA^−^ CCR7^−^), and Tcm (CD45RA^−^ CD62L^+^ or CD45RA^−^ CCR7^+^) cells were determined in both CD4^+^ and CD8^+^, whereas effector cells (CD45RA+ CD62L− or CD45RA+ CCR7−) were presented in only CD8^+^. Although the similarity of flow cytometric plots of T cell subsets using different mAb combinations was observed, the intensity of CD62L expression was slightly brighter.

**Figure 2:**
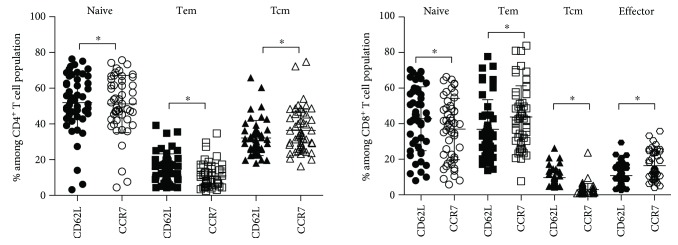
Comparison of T cell subsets of CD4^+^ and CD8^+^ T cells detected by using CD62L and CCR7. Line bar represents average ± SD; ∗indicates significant difference at *P* < 0.05.

With respect to cell number, the frequencies of T cell subsets of CD4^+^ and CD8^+^ from fifty HIV-infected children using the two different mAb sets were compared (Figure 2()). Naïve cells of CD4^+^ and CD8^+^ stained with CD62L were in greater amount than those stained with CCR7. In CD4^+^ population, the frequencies of Tem and Tcm cells from CD62L were higher and lower, respectively, than those from CCR7. These results of Tem and Tcm cells were vice versa in CD8^+^ population. A quantity of effector CD8^+^ T cells from CD62L was fewer than that from CCR7.

### 3.2. Effects of Viral Replication and Immunization on T Cell Subsets' Quantities

**Figure 3:**
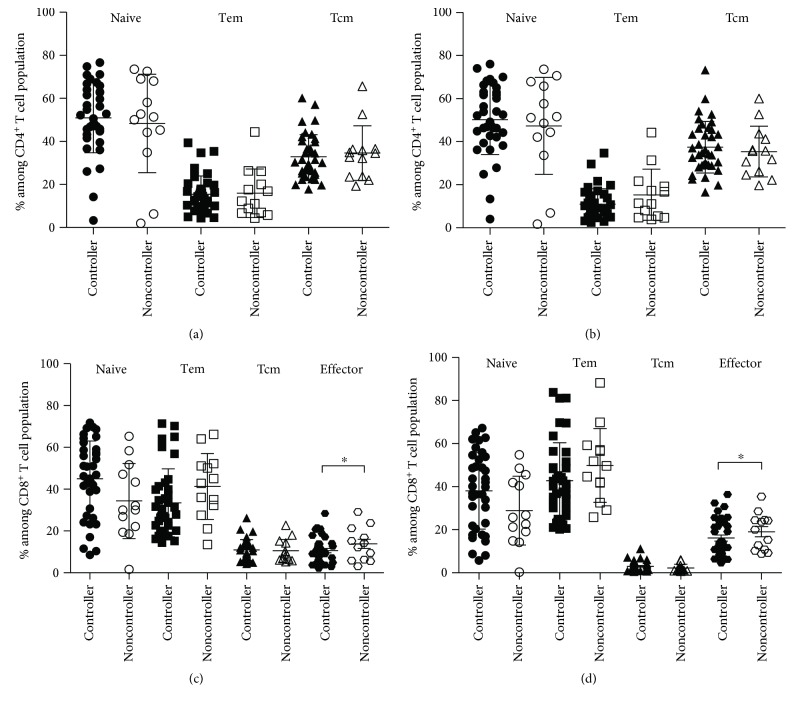
Comparison of T cell subsets between HIV-infected children with an undetectable viral load (controller group) and those with a virological failure (noncontroller group) detected by CD62L (a, c) and CCR7 (b, d). Line bar represents average ± SD; ∗indicates significant difference at *P* < 0.05.

To date, an influence of HIV viral replication on T cell subpopulations remains equivocal. To determine this, all HIV-infected patients receiving vaccination were examined for their viral load levels and then divided into controller and noncontroller groups. Frequencies of CD4^+^ T cell subsets of controller and noncontroller groups are compared in Figures 3(a) and 3(b) when using CD62L and CCR7, respectively. There was no difference between the two different groups as well as the two different staining sets. Frequencies of CD8^+^ T cell subpopulations of the two groups when using the two mAb combinations are also presented in Figures 3(c) and 3(d). The data shows the same trend when using CD62L and CCR7. All subpopulations of controller and noncontroller groups gave the similar numbers, except effector cells. Effector cells in the controller group had a lower amount than those in the noncontroller group (10.6% versus 13.8% when staining with CD62L and 16.1% versus 19.1% when staining with CCR7).

**Figure 4:**
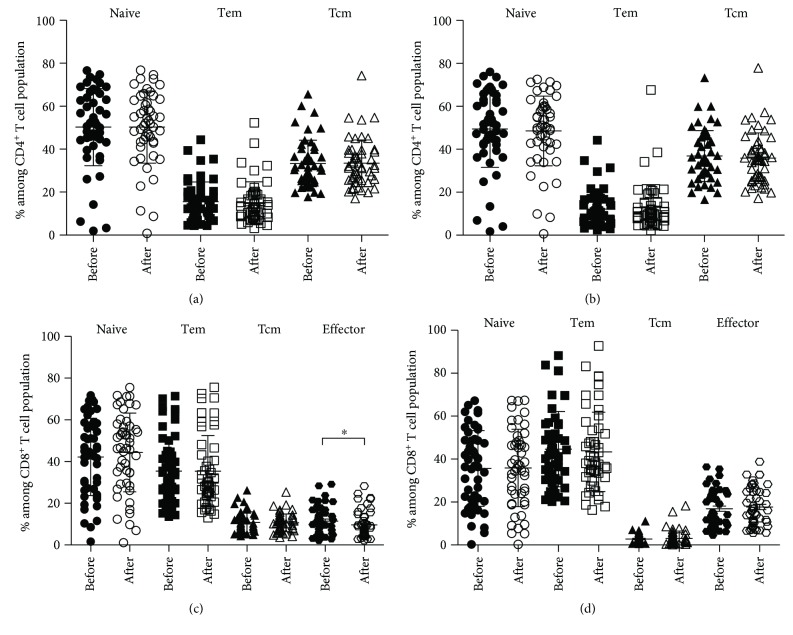
Comparison of T cell subsets before and after immunization in total population of HIV-infected patients detected by CD62L (a, c) and CCR7 (b, d). Line bar represents average ± SD; ∗indicates significant difference at *P* < 0.05.

To evaluate the impact of immunization on T cell distribution, the whole study population was examined at two time points, before and after inoculation. Frequencies of CD4^+^ T cell subpopulations when using CD62L and CCR7, respectively, are presented in Figures 4(a) and 4(b), and similarly for CD8^+^ T cell subsets in Figures 4(c) and 4(d). Naïve, Tem, and Tcm cells of both CD4^+^ and CD8^+^ showed no difference between before and after vaccination from the two mAb sets. Effector CD8^+^ T cells before immunization were in greater amount than those after immunization (11.5% versus 9.6%) when using CD62L. Effector CD8^+^ T cells before immunization were, however, similar to those after immunization when using CCR7. It is worth noting that using either CD62L or CCR7 did not make any difference to T cell subset distribution patterns.

Due to a significant decrease in effector CD8^+^ T cells after vaccination (Figure 4(c)), further investigation for the effect of viral replication was conducted in these effector CD8^+^ T cells. For the controller group, effector cells before vaccination with the amount of 10.6 ± 6.4% were reduced to 8.9 ± 6.0% after vaccination. The noncontroller group, however, had almost an identical profile of effector cells between before and after immunization (data not shown). Therefore, viral replication does not affect the change of effector cells.

In addition, frequencies and absolute counts of total CD4^+^ and CD8^+^ T cells were also compared (Table 1). There was no significant difference between before and after immunization in the controller group, noncontroller group, and total population.

### 3.3. Correlation between T Cell Subsets and CD4 Count

Correlations between CD4^+^ and CD8^+^ T cells (i.e., absolute CD4^+^ and CD8^+^ counts and their percentages) and their subsets (i.e., naïve, effector, Tem, and Tcm cells) from the whole subject population (*n* = 50) after immunization were analyzed and are shown in Table 2. A relationship of only absolute CD4 count with all CD4^+^ subsets and naïve CD8^+^ T cells was found. There were little differences of the data obtained from CD62L compared to CCR7.

## 4. Discussion

In order to identify T cell subpopulations, immunofluorescent staining with CD45RA and CD45RO has been commonly used to classify naïve and memory cells in CD4^+^ and CD8^+^. Sallusto et al. reported that memory CD8^+^ T cells were able to be further divided into Tcm and Tem cells by detecting expressions of two lymph node homing receptors of CD62L and CCR7 [12]. Tcm cells showed no expression in CD45RA and expressed both CD62L and CCR7 (CD45RA^−^ CD62L^+^ CCR7^+^), resulting in the cell capability of returning to the lymph node. Tem cells, on the other hand, did not express all those markers (CD45RA^−^ CD62L^−^ CCR7^−^), causing the lack of that cell ability and remaining in bloodstreams, spleens, and nonlymphoid tissues.

Our study distinguishes two important points on how the markers' utilization affects a detectable ability of memory T cell population when using CD62L and CCR7. The results firstly showed that frequencies of CD62L^+^ or CCR7^+^ in Tcm cells and CD62L^−^ or CCR7^−^ in Tem cells are not necessarily equal. In CD4^+^ population, Tem cells using CD62L had greater amount than those using CCR7 and Tcm cells showed the opposite outcomes. Tem and Tcm cells in CD8^+^ population were vice versa to those in CD4^+^. Secondly, Tcm cells in CD4^+^ and CD8^+^ populations can also be identified in more than one pattern when simultaneously stained with CD62L and CCR7. Tcm CD4^+^ cells were able to be characterized with the expressions of CD45RA^−^ CD62L^+^ CCR7^+^, CD45RA^−^ CD62L^+^ CCR7^−^, and CD45RA^−^ CD62L^−^ CCR7^+^, whereas identification of Tcm CD8^+^ cells showed the same patterns but excluding the latter (data not shown).

In an effort to understand the impact of viral replication and immunization on ART-treated HIV-infected children, the effect of ART itself on T cell subset distribution has to be primarily established. There are a few studies conducted in children, while most investigations were performed in adult patients. Chen et al. found that naïve (CD45RA^+^ CCR7^+^) and Tcm cells in CD8^+^ population dramatically reduced, whereas Tem cells increased in HIV-infected patients [13]. More evidence also supported the previous finding that Tem cells were also abundantly found in CD8^+^ T cells together with a low level of Tcm cells [14, 15]. As far as the efficacy of HAART is concerned, HAART inhibits viral replication and increases numbers of CD4^+^ T cells [16–18]. When further focusing on the change of T cell subsets, naïve CD4^+^ and CD8^+^ T cells increased and memory CD8^+^ T cells significantly decreased [19]. An increase in naïve CD8^+^ T cells and a decrease in memory CD4^+^ T cells were also observed in HIV-infected children receiving HAART for 44 weeks [20].

Our study concurs with the previous findings that naïve cells, followed by Tcm and Tem cells, were predominantly found in CD4^+^ T cells in HIV-infected children treated with ART. CD8^+^ population, however, showed differently as Tem cells were in majority, followed by naïve, effector, and Tcm cells.

Concerning viral replication affecting T cell subpopulations, Anselmi et al. found that only naïve T cells in HIV-infected children who had a virological failure after HAART initiation were higher than those in the patients who had a controllable viral load [21]. However, our results did not support that. We found no differences in frequency of all CD4^+^ and most CD8^+^ T cell subsets (i.e., naïve, Tem, and Tcm cells) between the controller (having a controllable viral load, <40 copies/mL) and noncontroller (having a virological failure, ≥40 copies/mL) groups. Only the frequency of effector CD8^+^ T cells in the controller group was higher than that in the noncontroller group. We then suggest that a transient increase in viral replication does not significantly change naïve, effector, and memory T cells.

When the influenza vaccine was inoculated, Gunthard et al. found that naïve CD4^+^ T cells transiently decreased and activated memory CD4^+^ T cells increased in healthy volunteers [22]. For HIV-infected patients, on the other hand, naïve cells remained the same and activated memory T cells were reduced before returning to the baseline after 8 weeks. Another investigation also reported that naïve and memory T cells had no significant difference when inoculated with tetanus vaccines [23]. Our study complies with the previous findings as we found only effector CD8^+^ T cells notably decreased in the controller group after influenza vaccination and no change in the noncontroller group. It is then suggested that viral replication has no influence on the alteration of T cell subsets in ART-treated HIV-infected children after immunization. Likewise, immunization with the influenza A (H1N1) 2009 vaccine does not affect T cell subset distribution in ART-treated HIV-infected children.

We also verified the correlation between T cell subset frequencies and absolute counts of CD4 and CD8. We found a positive relationship of absolute CD4 count with naïve CD4^+^ and CD8^+^ T cells, suggesting that ART-treated patients with a high CD4^+^ T cell level are able to maintain a high naïve T cell level. However, the origin of naïve T cells remains unclear whether derived from new generation of T cells or redistributed from somewhere else. In contrast, our result showed a negative correlation between absolute CD4 count and Tcm cells, which differs from a study showing that Tcm cells were maintained in patients who can control viral replication without any antiretroviral treatment [24]. These different observational patterns suggest different mechanisms controlling T cell subset dynamic between antiretroviral-treated patients and patients who can naturally control viral replication.

## 5. Conclusions

This is the investigation of the impact of viral replication and immunization on individual naïve, effector, effector memory, and central memory T cell subpopulations from both CD4^+^ and CD8^+^ T cells in ART-treated HIV-infected children. Naïve cells were predominant in CD4^+^ T cells, whereas effector memory cells were mainly found in CD8^+^ T cells in the patients without vaccination. No significant difference was observed between the patients with controllable and noncontrollable viral loads, except effector CD8^+^ T cells, suggesting that a transient increase in viral replication does not affect the T cell distribution. This is also confirmed by the results after inoculation of the influenza A (H1N1) 2009 vaccine, which shows that T cell distribution did not change in the patients with virological failure. Immunization also did not affect all T cell subpopulations, except effector CD8^+^ T cells in the patients with controllable viral loads. Therefore, our finding can ensure physicians that the immunization of influenza A (H1N1) 2009 vaccine is safe to be used together with ART in HIV-infected children. Moreover, a correlation between T cell subset frequencies and absolute counts of CD4 and CD8, which are generally observed for disease progress, has been verified. Only naïve CD4^+^ and CD8^+^ T cells had a positive relationship with absolute CD4 count. The classical CD4 count can thus be useful for prediction of naïve T cell level in HIV-infected patients responding to ART.

## Figures and Tables

**Table 1:** Percentages of CD4 and CD8 and absolute CD4 and CD8 counts before and after immunization in the controller and noncontroller groups and total population.

Group	Controller group (*n* = 37)		Noncontroller group (*n* = 13)		Total population (*n* = 50)	
	Before immunization	After immunization	Before immunization	After immunization	Before immunization	After immunization
% CD4	29.3 ± 9.0	30.7 ± 8.5	25.7 ± 12.3	25.9 ± 14.2	28.4 ± 9.9	29.5 ± 10.3
Absolute CD4 count (cells/*μ*L)	982 ± 539	1008 ± 585	1073 ± 992	1082 ± 914	1006 ± 675	1027 ± 676
% CD8	39.4 ± 10.0	38.6 ± 9.0	38.6 ± 10.1	41.0 ± 12.8	39.2 ± 10.0	39.3 ± 10.0
Absolute CD8 count (cells/*μ*L)	1255 ± 478	1219 ± 546	1322 ± 526	1478 ± 573	1273 ± 487	1286 ± 559

Note: data are shown as average ± SD.

**Table 2:** Correlations between each of T cell subsets and percentages of CD4 and CD8 and absolute CD4 and CD8 counts in ART-treated HIV-infected children after immunization (*n* = 50).

Marker	T cell subset	Correlation (*r*)			
		Versus	Versus	Versus	Versus
		% CD4	absolute CD4 count	% CD8	absolute CD8 count
CD45RA/CD62L	Naïve CD4^+^ T cells	0.6427^∗^	0.7127^∗^	−0.5365^∗^	0.1302
	Tem CD4^+^ T cells	−0.6430^∗^	−0.7190^∗^	0.6179^∗^	−0.1103
	Tcm CD4^+^ T cells	−0.4515^∗^	−0.5547^∗^	0.3462^∗^	−0.1627

CD45RA/CD62L	Naïve CD8^+^ T cells	0.5579^∗^	0.5048^∗^	−0.5677^∗^	−0.1335
	Effector CD8^+^ T cells	−0.1930	−0.0072	0.2275	0.3175^∗^
	Tem CD8^+^ T cells	−0.5008^∗^	−0.4741^∗^	0.5756^∗^	0.0858
	Tcm CD8^+^ T cells	0.0232	−0.0745	−0.1979	-0.2180

CD45RA/CCR7	Naïve CD4^+^ T cells	0.6865^∗^	0.7184^∗^	−0.5362^∗^	0.0787
	Tem CD4^+^ T cells	−0.7583^∗^	−0.6958^∗^	0.6892^∗^	0.0806
	Tcm CD4^+^ T cells	−0.3670^∗^	−0.4740^∗^	0.2817^∗^	−0.1466

CD45RA/CCR7	Naïve CD8^+^ T cells	0.5554^∗^	0.4272^∗^	−0.5223^∗^	−0.2269
	Effector CD8^+^ T cells	−0.0085	0.1533	−0.0547	0.2947^∗^
	Tem CD8^+^ T cells	−0.5335^∗^	−0.4887^∗^	0.5583^∗^	0.1208
	Tcm CD8^+^ T cells	0.3298^∗^	0.3430^∗^	−0.3485^∗^	−0.0199

Note: ^∗^significant difference at *P* < 0.05.
